# Left atrial epicardial adipose tissue volume quantification by CMR aids identification of patients at low risk for left atrial cardiomyopathy

**DOI:** 10.1007/s00392-025-02718-0

**Published:** 2025-09-03

**Authors:** T. R. Schmidt, S. Ulbrich, T. Gaspar, M. Wagner, S. Richter, A. Linke, F. M. Heidrich

**Affiliations:** Department for Internal Medicine and Cardiology, Heart Center Dresden, Faculty of Medicine and University Hospital Carl Gustav Carus, TUD Dresden University of Technology, Dresden, Germany

**Keywords:** Left atrium, Epicardial adipose tissue, Left atrial cardiomyopathy, Low-voltage zone, Prediction model, Volume quantification

## Abstract

**Background and aims:**

The pathophysiologic concept of atrial fibrillation (AF) has evolved towards defining atrial cardiomyopathy, recognizing inflammation-mediated remodeling of the left atrium (LA) as a source for arrhythmogenesis. One feature of atrial cardiomyopathy is the development of fibrosis, with low-voltage zones (LVZ) identified by invasive electroanatomic mapping as an accepted surrogate parameter. A mediator of pathological remodeling is epicardial adipose tissue (EAT). This study sought to explore LA-EAT volume, as a predictor of LVZ in patients presenting for primary AF ablation.

**Methods and results:**

CMR imaging of left atrial epicardial adipose tissue was performed using fat–water separation Dixon-based sequences in 58 patients (mean age 68.2 ± 10.1 years) presenting for primary pulmonary vein isolation (PVI). Additionally, left atrial volume index (LAVi) was derived from contrast-enhanced angiography. Left atrial epicardial volume index (LA-EATVi) was a significant predictor of LVZ, with significantly higher volumes in LVZ + patients (mean difference of 7.2 ± 2.4 ml/m^2^), a moderate correlation (*r* = 0.37, *p* < 0.001), and a univariate predictive ability with an area under the curve (AUC) of 0.71. Expanding the prediction model with age, gender, and LAVi improves the prediction of LVZ up to an AUC of 0.91. Cutoff selection at 0.25 predicted probability identifies a low-risk group for LVZ, with a negative predictive value of 96.7%, sensitivity 95%, and specificity 76.3%.

**Conclusion:**

The pre-procedural identification of a low risk of atrial cardiomyopathy is important to select patients for single-shot catheter ablation. LA-EATVi proved to be of additive value to known risk factors for the prediction of LVZ in a combined prediction model. Patients not considered low risk could be offered an electroanatomic atrial mapping for LVZ detection with the possibility of an LVZ-based radiofrequency ablation approach. Individualized matching of patient and ablation technique using an LVZ prediction model might lead to improved ablation outcomes.

**Graphical Abstract:**

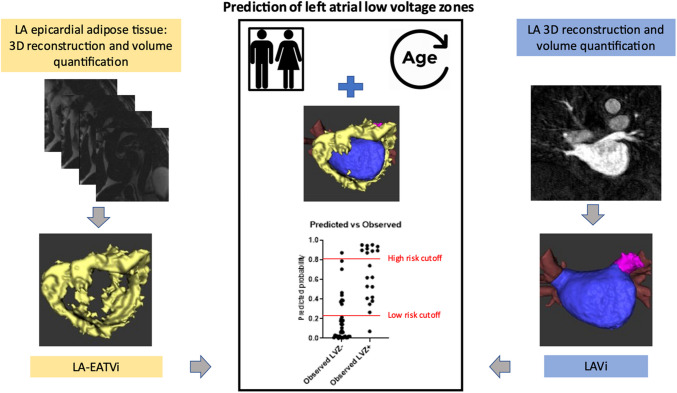

**Supplementary Information:**

The online version contains supplementary material available at 10.1007/s00392-025-02718-0.

## Introduction

The current concept of left atrial cardiomyopathy comprises a combination of cardiomyocyte and fibroblast dysfunction, with collagen and non-collagenous deposition, like inflammatory and fatty infiltrates in the left atrial wall, contributing to fibrosis [1]. Invasive endocardial voltage mapping–derived low-voltage zones (LVZ) are an accepted surrogate parameter for left atrial fibrosis [2].

Increasing knowledge of inflammatory processes and fatty infiltration expands our understanding of the pathophysiology of atrial cardiomyopathy and atrial fibrillation (AF) [3]. Evidence from experimental studies in a sheep model shows that obesity was associated with reduced posterior LA endocardial voltage and continuous infiltration of posterior LA myocardium by epicardial adipose tissue (EAT) [4]. Additionally, a study using human EAT taken from aorto-coronary bypass patients could prove the induction of atrial fibrosis by adipo-fibrokines such as Activin A in an organo-culture model of rat atria [5]. Histologic evidence of the pathophysiologic concept has been shown by a study of surgically obtained LAA samples. Different EAT remodeling causing profiles were associated with LA myocardial fibrosis as assessed by microscopic examination, with the severely fibrotic EAT profile showing the most abundant collagen fiber accumulation as well as the presence of stromal cells (macrophages and myofibroblasts). The authors further describe the infiltration of EAT into the atrial myocardium and the resulting atrial myocardial fibrosis occurring primarily at the junction of these two structures. Further, the total collagen in the LA myocardium was positively correlated with proinflammatory and pro-fibrotic cytokines/chemokines, including interleukin-6, monocyte chemoattractant protein-1, and tumor necrosis factor-α in EAT [6].

EAT displays unique tissue characteristics and emerges as pivotal in arrhythmogenesis and inflammatory processes, as it is in direct contact with the underlying myocardium and shares the vascular supply [4, 7–10]. EAT displays an intermediate beige phenotype with tissue plasticity and features characteristic of both white and brown adipose tissue [11–14]. The beige phenotype is not a permanent tissue state; it is an inducible characteristic (via transdifferentiation from a white phenotype by “browning”) [9, 15–17]. It has been demonstrated that this plasticity of beige adipose tissue is lost with higher age, advanced coronary artery disease, and especially in female patients, favoring a transdifferentiation towards the white adipose tissue phenotype [13, 18–22].

The secretome of white phenotype EAT contains molecules with proinflammatory effects, which lead to the activation of the innate and adaptive immune systems and infiltration of tissues with proinflammatory macrophages among many others. Additionally, bioactive molecules lead to the remodeling of the extracellular matrix and fibrogenesis. These processes create a complex state of adipose tissue inflammation [9, 13, 23, 24].

The role of EAT in the development of atrial cardiomyopathy, arrhythmogenesis, and AF has been described, and a number of studies have correlated the described mechanisms with a higher local EAT volume [7, 24–27]. Additionally, the loss of the beige phenotype has been identified as a driver of pathological processes [28].

AF, as the most common sustained cardiac arrhythmia, is a challenge to public health due to major cardiovascular morbidity and increased mortality [29, 30]. Current treatment strategies favor the restoration of sinus rhythm either by catheter ablation or antiarrhythmic medication [31, 32]. Evidence shows that catheter ablation in selected AF patients with heart failure with reduced left ventricular ejection function leads to a reduction of all-cause mortality, cardiovascular-related mortality, and hospitalizations [33–36]. Catheter ablation is currently recommended as a first-line therapy for all symptomatic patients with paroxysmal AF, with superior results for long-term rhythm control [37].

The choice of treatment approach should be individualized and aimed to reach a long-term reduction of AF burden. Strategies including extended ablation of extra-pulmonary arrhythmogenic areas, identified as low-voltage zones (LVZ), reduce the risk of AF recurrence in patients with atrial cardiomyopathy [38]. Up until now, the prediction scores for the presence of LVZ, like APPLE or mAPPLE, take into account patient characteristics and left atrial size [39, 40].

The current study aims to correlate left atrial EAT volume (LA-EATV) assessed by CMR to the presence of left atrial LVZ, as a surrogate parameter of LA fibrosis and atrial cardiomyopathy to establish a prediction model for LVZ.

## Methods

A single-center prospective exploratory observational study was performed. Patients at the University Heart Center Dresden admitted for catheter ablation of symptomatic atrial fibrillation underwent a routine pre-procedural CMR for ablation planning. Consecutive patient data was collected from April 2019 until August 2020. The study was approved by the local ethics committee (EK 284092012). Informed patient consent was obtained.

### Inclusion and exclusion criteria

Inclusion criteria for this study were symptomatic paroxysmal or persistent AF, first AF ablation procedure, assessment of left atrial bipolar voltage mapping during AF ablation in sinus rhythm, and pre-procedural CMR imaging. All patients admitted for primary AF ablation at our center were eligible for a pre-procedural CMR to evaluate the pulmonary vein anatomy. However, due to limited CMR capabilities, not all patients received a CMR prior to the procedure or did not receive additional adipose tissue image acquisition, which was an inclusion criterion for this study. No clinical criteria were applied to select patients for CMR to avoid a selection bias. Re-do procedures did not routinely receive CMR imaging since either a previous CT angiography was present or an electro-anatomical map was available from the previous ablation.

**Fig. 1:**
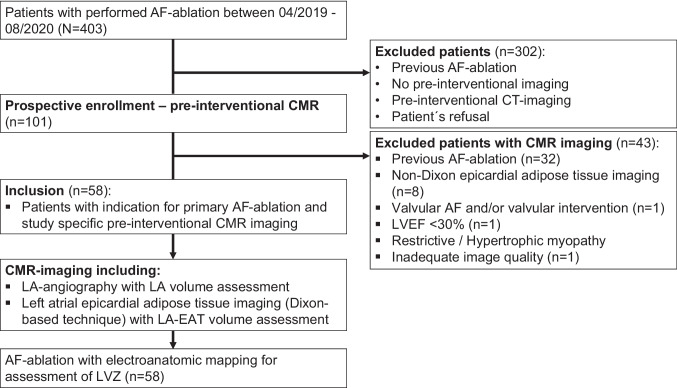
Study design flowchart. Abbreviations: atrial fibrillation (AF), cardiac magnetic resonance (CMR), left atrium (LA), left atrial appendage (LAA), low-voltage zones (LVZ), left ventricular ejection fraction (LVEF), Dixon imaging as first described fat–water separation imaging by W. T. Dixon in 1984

Patients were excluded from this study if permanent or valvular AF, any previous AF ablation, left ventricular ejection fraction <30%, corrected mitral valve disease, restrictive or hypertrophic cardiomyopathy, or left atrial appendage thrombus was present. Additionally, patients were excluded with missing or non-Dixon epicardial adipose tissue imaging or inadequate image quality. A study protocol overview is provided in Fig. 1.

### CMR protocol and volume quantification

The CMR protocol (Siemens Healthineers MAGNETOM Aera 1.5T) consisted of a gadolinium-based 3D contrast-enhanced MR angiography with real-time bolus tracking to evaluate left atrial and pulmonary vein anatomy, using a FLASH3D gradient echo sequence (single breath-hold, real-time bolus tracking, without ECG gating) for image acquisition with the following parameters: FOV 400 mm, matrix 269 pixels × 384 pixels, voxel size 0.5 mm × 0.5 mm × 1 mm.

**Fig. 2:**
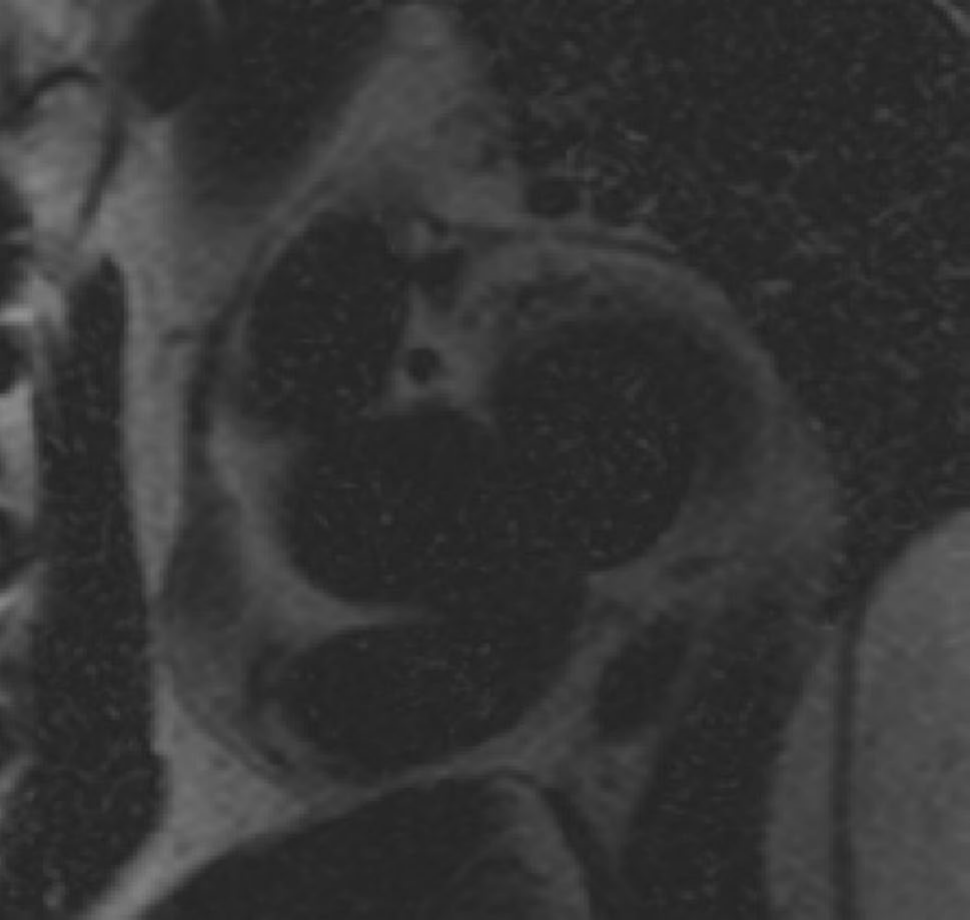
Example of a short-axis fat-only Dixon image of epicardial adipose tissue on the level of the left atrium, dorsal to the mitral valve plane

Adipose tissue adjacent to the left atrium was visualized using a fat-water separation sequence based on the Dixon imaging method, resulting in fat-only images (Fig. 2) [41, 42]. Imaging parameters used were slice thickness 5–7 mm, spacing between slices 5–8 mm, FOV 420 mm, matrix 144 pixels × 256 pixels, voxel size 0.8 mm × 0.8 mm × 5–7 mm, TR 586 ms, TE 1.5 ms. The number of acquired short-axis slices ranged from 6 to 10 to cover the whole left atrium of varying dimensions.

Generation of 3D models and volume quantification were performed using ADAS 3D software [43] (Galgo Medical S. L, 2019). The functionalities include 3D left atrial semiautomatic segmentation and automatic classification of tissues using selectable imaging thresholds. An example segmentation workflow is shown in the supplement.

### Assessment of low-voltage zones

Presence and extent of LVZ were assessed during the pulmonary vein isolation procedure by bipolar voltage mapping of the left atrial wall using a multipolar mapping catheter of variable diameter size (15–25 mm) and 1 mm electrode/8 mm spacing (Lasso 2515 NAV Eco, Biosense Webster, Inc., Diamond Bar, CA, USA, or Advisor™HD-Grid, Abbott, Abbott Park, IL, USA). All point measurements were taken during sinus rhythm. Patients that presented with AF received an electrical cardioversion during the procedure. In case of unsuccessful initial cardioversion, isolation of the pulmonary vein ostia was performed and the cardioversion attempt repeated. Classification of a prespecified left atrial wall region as being a low-voltage zone (LVZ) was performed if a median voltage ≤0.5 mV was recorded [44].

### Statistics

Statistical analyses were conducted using IBM SPSS (version 26; IBM Corp., Armonk, NY, USA) and GraphPad Prism (version 9.4.0; GraphPad Software, San Diego, CA, USA). Continuous variables were tested for normality with the D’Agostino–Pearson test. Normally distributed data are reported as mean ± standard deviation (SD), while non-normal data are presented as median (interquartile range). Between-group comparisons of continuous variables employed the Student’s *t*-test or Mann–Whitney *U* test, as appropriate. Categorical variables are expressed as counts and percentages and compared using Fisher’s exact test.

Correlations between two normally distributed continuous variables were assessed by Pearson’s correlation coefficient; point-biserial correlation was used when one variable was dichotomous. Associations between two binary variables (e.g., gender and LVZ presence) were estimated using the tetrachoric correlation coefficient under a bivariate normal model. Robust 95% confidence intervals and two-tailed *p*-values were derived from 1000 bootstrap resamples.

Intra-observer and inter-observer reliability for imaging measurements was evaluated using intraclass correlation coefficients (ICCs). Intra-rater reliability for CMR-derived left atrial volume (LAV) and left atrial epicardial adipose tissue volume (LA-EATV) was assessed by having the same investigator remeasure absolute values in ten randomly selected patients, blinded to prior results and clinical data, at two time points approximately 3 months apart. Inter-observer reliability for LA-EATV was assessed by a second investigator in 12 randomly selected patients, blinded to previous results. The ICC (95% CI) was calculated from single measurements using a two-way mixed-effects model, absolute agreement definition. For inter-observer reliability, a secondary analysis of consistency was performed. ICC values were interpreted according to conventionally accepted thresholds [45].

Prior to regression modeling, multicollinearity among predictor variables was examined by calculating variance inflation factors (VIFs), with VIF >5 indicating potential collinearity concerns.

Predictive models were developed via binomial regression including main effects only, and their discriminative performance was assessed by receiver-operating characteristic (ROC) analysis. Regression coefficient stability was validated by bootstrap resampling (*n* =2000) to derive bias-corrected and accelerated confidence intervals.

Model improvement was quantified using the categorical net reclassification index (NRI) with predefined risk categories (< 10%, 10–20%, > 20%). The NRI reflects the proportion of events correctly reclassified upward and non-events correctly reclassified downward by the new model versus the reference. Ninety-five percent confidence intervals and *p*-values for the NRI were obtained via 1000 bootstrap replicates.

Unless otherwise specified, statistical significance was defined as *p* <0.05. In the binomial regression, predictors with *p* ≤0.20 were retained for model building.

## Results

### Patient characteristics

In this analysis, 58 patients met the inclusion criteria. The general patient characteristics are displayed in Table 1.

**Table 1:** Baseline characteristics of the studied patient population.

Variable	Patient population (*n* = 58)	LVZ + (*n* = 20)	LVZ- (*n* = 38)	*p*-value
Gender (male), *n* (%)	34 (59)	6 (30)	28 (74)	0.002
Age (years)	68.2 ± 10.1	74.2 ± 6.5	65.1 ± 10.2	< 0.001
BMI (kg/m^2^)	27.6 (25.5–30.7)	28.6 (25.0–30.6)	27.4 (25.5–31.1)	0.89
LVEF (%)	60 (56–64)	61.0 ± 10.5	59.0 ± 6.0	0.38
Persistent AF, *n* (%)	36 (62)	14 (70)	22 (58)	0.41
Arterial hypertension, *n* (%)	51 (88)	18 (90)	33 (87)	0.99
Cerebrovascular incident, *n* (%)	7 (12)	2 (10)	5 (13)	0.99
Coronary artery disease, *n* (%)	11 (19)	5 (25)	6 (16)	0.49
Diabetes mellitus type 2, *n* (%)	10 (17)	5 (25)	5 (13)	0.29
Dyslipidemia, *n* (%)	30 (52)	9 (45)	21 (55)	0.58

Abbreviations: BMI body mass index, LVEF left ventricular ejection fraction, AF atrial fibrillation

### CMR-derived parameters

Evaluated CMR parameters of left atrial volume and left atrial epicardial adipose tissue volume were indexed to body surface area (BSA), calculated by the Mosteller method [46]. Results are displayed for both analyzed patient groups regarding their LVZ status and show a larger LAVi and LA-EATVi in LVZ+ patients (Table 2).

**Table 2:** CMR-derived variables of left atrial volume index (LAVi) and left atrial epicardial adipose tissue volume index (LA-EATVi) according to low-voltage zone (LVZ) status

Variable	LVZ + (*n* = 20)	LVZ- (*n* = 38)	Difference between means	95% CI	*p*-value
LAVi (ml/m^2^)	75.0 ± 19.2	58.8 ± 15.1	16.2 ± 4.6	7.0‒25.4	< 0.001
LA-EATVi (ml/m^2^)	32.3 ± 10.1	25.1 ± 7.8	7.2 ± 2.4	2.4‒11.9	0.004

Intra-rater reliability for CMR-derived LAV and LA-EATV (*n* =10), measured by one blinded observer at two time points ~3 months apart, was excellent (ICC for LAVi 0.98 95% CI (0.91–1.00) and for LA-EATVi 0.94 95% CI (0.76–0.98)) as can be seen in Table 3.

**Table 3:** Intraclass correlation coefficients (ICC) for CMR-derived LAV and LA-EATV (*n* = 10), calculated using a two-way mixed-effects, single-measurement, absolute agreement model. ICC (95% CI) values < 0.50 indicate poor reliability, 0.50–0.75 moderate, 0.75–0.90 good, and > 0.90 excellent [45].

Variable	ICC	95% CI	*p*-value
LAV	0.98	0.91‒1.00	< 0.001
LA-EATV	0.94	0.76‒0.98	< 0.001

Inter-rater reliability for absolute agreement of CMR-derived LA-EATV (*n* =12) was measured by a second investigator, blinded to previous measurements. The results indicate a good reliability with ICC of 0.75 (95% CI (0.47–0.91)), *p* <0.001. A secondary ICC analysis for consistency yielded a slightly higher value of 0.78 (95% CI (0.51–0.92)), *p* <0.001.

### LVZ prediction models

After initial analysis of variables from patients’ characteristics and CMR, their correlation with the dichotomous outcome variable (LVZ) using point-biserial correlation was assessed. The results are displayed in Table 4. It is worth mentioning that body mass index (BMI) as a universal marker of adiposity did not correlate with LA-EATVi (*r* =−0.06 *p* =0.64), LAVi (*r* =−0.18 *p* =0.17), nor LVZ occurrence (*r* =0.06 *p* =0.7). The two binary variables gender and LVZ correlated strongly using tetrachoric correlation (*r*_tet_ =0.63 *p* <0.001).

**Table 4:** Correlation coefficients (*r*) between selected predictors and the presence of a low-voltage zone (LVZ; dichotomous outcome). Continuous variables included age, body mass index (BMI), left atrial volume index (LAVi), and left atrial epicardial adipose tissue volume index (LA-EATVi). For the binary variable gender, *r* was estimated using tetrachoric correlation

Variable	*r*	95% CI	*p*-value
Age	0.43	0.20–0.62	< 0.001
Female gender	0.63	0.27–0.88	< 0.001
BMI	− 0.05	− 0.31–0.21	0.7
LAVi	0.43	0.19‒0.62	< 0.001
LA-EATVi	0.37	0.13‒0.58	< 0.001

After confirmation of a significant correlation with the outcome variable, prediction models were established using binomial logistic regression. This analysis estimates coefficients for each predictor and thereby describes their influence on the outcome risk estimation. The results for the univariate prediction models are reported in Table 5.

**Table 5:** Univariate binomial logistic regression for low-voltage zone (LVZ) prediction using a 0.5 probability cutoff (all *p* < 0.05). Reported metrics include *G*^2^ (log-likelihood ratio), Nagelkerke’s *R*^2^, AUC, and odds ratios (ORs) with 95% CIs

Variable	*G*^2^	*R*^2^	AUC	*p*-value	95% CI for AUC	OR	95% CI for OR	Sensitivity %	Specificity %
Age	12.83	0.27	0.76	0.001	0.64‒0.88	1.13	1.05‒1.24	50	81.6
Gender (female)	10.44	0.23	0.72	0.007	0.58–0.86	6.53	2.06‒23.2	70	73.7
LAVi	10.93	0.24	0.74	0.003	0.61–0.87	1.06	1.02‒1.10	30	82.6
LA-EATVi	8.47	0.19	0.71	0.008	0.57–0.86	1.10	1.03‒1.19	30	92.1

All four variables reached statistical significance in univariate analysis (Table 5) and were included in a multivariate prediction model in a forward stepwise approach:

$$LVZoccurrence=-14.07+0.08\times age+2.28\times femalegender+0.05\times LAVi+0.12\times LAEATVi.$$

**Fig. 3:**
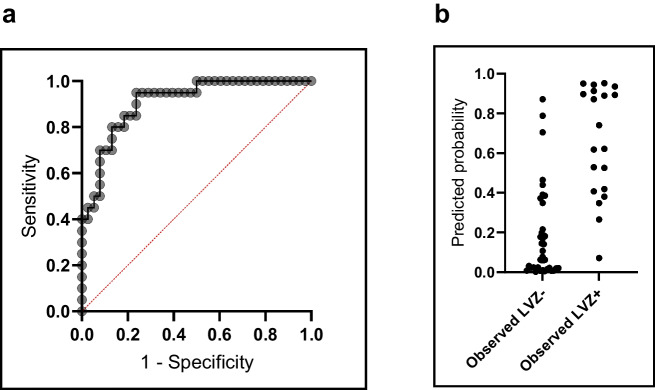
**a** ROC curve for the combined CMR-enhanced prediction model statistically described in detail in Table 6. **b** Predicted vs observed LVZ status using the combination of age, gender, left atrial volume index (LAVi), and left atrial epicardial adipose tissue volume index (LA-EATVi) as variables in the binomial regression equation

Table 6 and Figure 3 illustrate the performance of this prediction model. The model displays a high discrimination ability (AUC 0.91) and a high model fit (*G*^2^ 32.2) in calibration testing. Additionally, it shows a high ability to explain the variability of the outcome (*R*^2^ 0.59). We assessed multicollinearity between predictor variables (age, gender, LAVi, and LA-EATVi) using the variance inflation factor (VIF) and tolerance statistics. All VIFs were below 1.5 (maximum, 1.45 for age; minimum, 1.06 for LA-EATVi), indicating low collinearity. Additionally, the pairwise correlation between LAVi and LA-EATVi was *r* =0.22 (*p* =0.07), suggesting no strong linear relationship.

**Table 6:** Key statistics for the CMR-enhanced multivariate LVZ prediction model versus a non-CMR model. The CMR model incorporated age, gender, left atrial volume index (LAVi), and left atrial epicardial adipose tissue volume index (LA-EATVi); all *β* coefficients were retained at *p* ≤ 0.20. Models used a 0.5 probability threshold. Discrimination (AUC) was highly significant (*p* < 0.001) for both models. Calibration was assessed by the log-likelihood ratio test (*G*^2^) and explained variance by Nagelkerke’s *R*^2^. Odds ratios (ORs) with 95% CIs are reported

Variable combination	*β* coefficient	*p*-value	OR	95% confidence interval for OR	*G*^2^	*R*^2^	AUC	Sensitivity %	Specificity %
Age	0.08	0.19	1.08	0.97‒1.23	32.2	0.59	0.91*	70.0	92.1
Gender (female)	2.28	0.01	9.78	1.85‒75.70					
LAVi	0.05	0.06	1.05	1.00‒1.12					
LA-EATVi	0.12	0.01	1.13	1.04‒1.26					
Age	0.10	0.02	1.10	1.02–1.21	16.6	0.34	0.81*	50	86.8
Gender (female)	1.29	0.05	3.60	0.99–13.89

The validation of the individual predictors and the combined model using bootstrap is shown in the supplement (Tables S1 and S2). Additionally, the performance of other prediction models combining different variables is available in the supplement (Table S3).

We compared a simple clinical risk prediction model using only age and gender with the final CMR-enhanced prediction model (including LAVi and LA-EATVi) using net reclassification index and applying typical clinically relevant risk categories (<10%, 10–20%, >20%). The categorical NRI was 0.37 (95% CI 0.12–0.60; *p* =0.003). Among events, 22.7% were reclassified upward and 9.1% downward. Among non-events, 38.9% were reclassified downward and 11.1% upward. This indicates that the CMR-enhanced model meaningfully improved the assignment of patients to more appropriate risk categories, particularly by reclassifying non-events into lower risk categories.

**Fig. 4:**
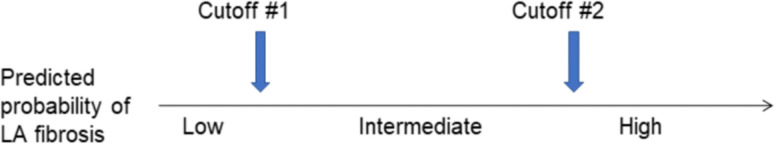
Scheme of cutoff to create three groups of patients by their predicted probability of left atrial (LA) fibrosis. Division into low, intermediate*,* and high probability groups

For clinical application of the proposed CMR-enhanced prediction model, cutoff selection was warranted. Cutoff values for risk classification were determined based on the ROC curve analysis to balance sensitivity and specificity according to clinical priorities (Fig. 4). The primary aim was to identify a low-risk group with high confidence, which required maximizing negative predictive value (NPV) and sensitivity to ensure that patients at low risk for LVZ were correctly identified for potential streamlined ablation strategies. For this purpose, a cutoff of 0.25 predicted probability was selected, which achieved a sensitivity of 95% and an NPV of 96.7% in our cohort (Table 7).

**Table 7:** Classification table of the combined prediction model for low-voltage zones (LVZ), including variables age, gender, left atrial volume index (LAVi), and left atrial epicardial adipose tissue volume index (LA-EATVi) with a cutoff set at 0.25 predicted probability

	Predicted LVZ-	Predicted LVZ +	Total	Correctly classified %
Observed LVZ-	29	9	38	76.32
Observed LVZ +	1	19	20	95.00
Total patients	30	28	58	82.76
Predictive value %	96.7	67.9		

Conversely, to identify a high-risk group with high specificity—prioritizing positive predictive value (PPV) to minimize false positives—a cutoff of 0.8 predicted probability was used. This threshold provided a PPV of 90% and a specificity of 97.4% (Table 8).

**Table 8:** Classification table of the combined prediction model for low-voltage zones (LVZ) including variables age, gender, left atrial volume index (LAVi), and left atrial epicardial adipose tissue volume index (LA-EATVi) with a cutoff set at 0.8 predicted probability

	Predicted LVZ-	Predicted LVZ +	Total patients	Correctly classified %
Observed LVZ-	37	1	38	97.37
Observed LVZ +	11	9	20	45.00
Total patients	48	10	58	79.31
Predictive value %	77.1	90		

**Fig. 5:**
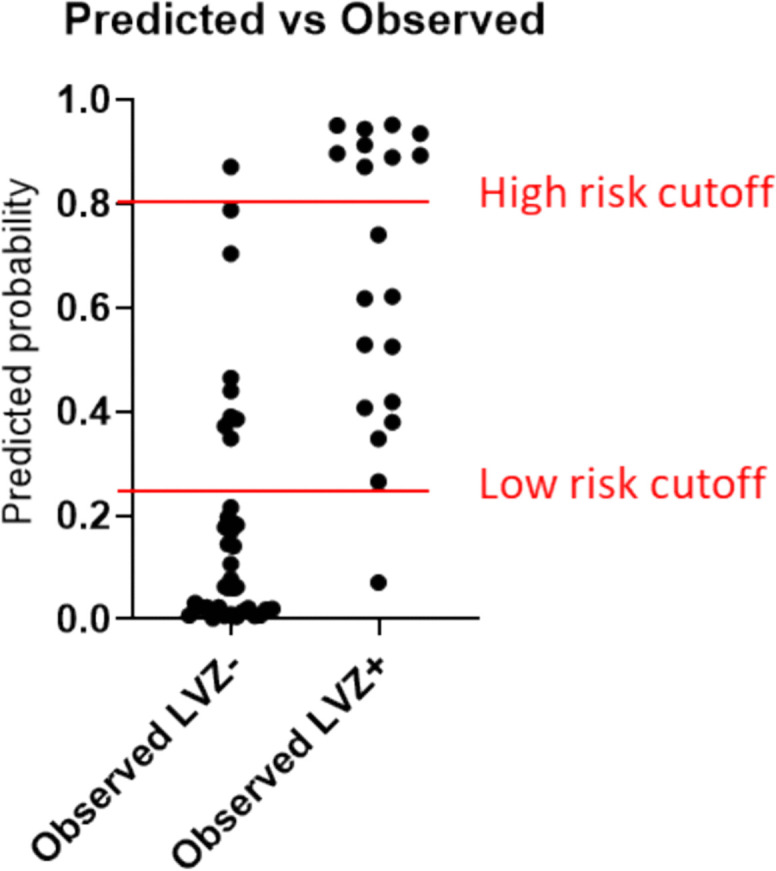
Visualization of cutoffs in the studied patient population. High-risk cutoff at 0.8 predicted probability and low-risk cutoff at 0.25 predicted probability using the combined prediction model of four variables (age, gender, left atrial volume index (LAVi), and left atrial epicardial adipose tissue volume index (LA-EATVi). Abbreviations: LVZ, low-voltage zone

The selection of two cutoffs enables us to distinguish three groups of patients as seen in Figs. 4 and 5.

## Discussion

This research demonstrates that quantification of left epicardial adipose tissue using Dixon-based CMR imaging aids prediction of low-voltage zones (LVZ) in the left atrium. Patients with LVZ show a significantly higher left atrial epicardial adipose tissue volume index (LA-EATVi) than patients without LVZ.

To the best of our knowledge, this work represents the first CMR Dixon-based approach, using dedicated adipose tissue imaging and analysis of the association between LA-EAT and invasive voltage measurements, describing LVZ as an electrophysiological surrogate for fibrosis of the atrial wall, which is a feature of left atrial cardiomyopathy. One previous CMR study used cine sequences to estimate adipose tissue volumes adjacent to the left atrium [7]. The presented results of our study are in agreement with a previous investigation of LA-EAT volume using CT-based assessment, which demonstrated the predictive ability regarding LVZ detection [47].

Epicardial adipose tissue has been previously associated with AF occurrence using a Dixon-based CMR assessment [48–50]. During our research study, we could also confirm age, female gender, and increased left atrial volume index (LAVi) as predictors of LVZ [39, 40, 44].

LAVi and LA-EATVi were not linearly correlated to each other and were able to independently predict LVZ (i.e., atrial fibrosis) in our study.

**Fig. 6:**
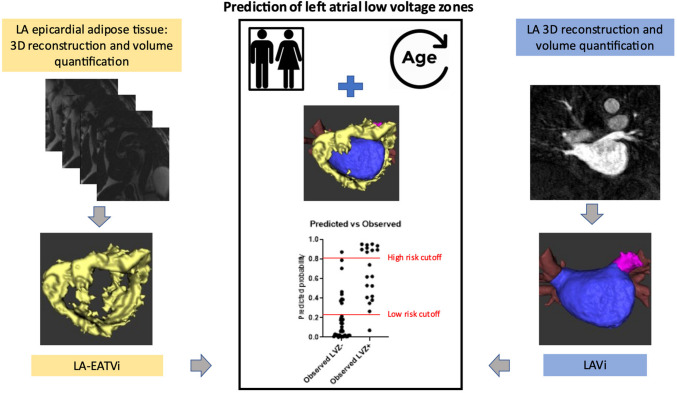
Central illustration: Combining two CMR imaging approaches for prediction of low-voltage zones (LVZ) in the left atrium. Assessment of left atrial epicardial adipose tissue volume index (LA-EATVi) using the Dixon method for generation of a short-axis stack covering the left atrium*,* resulting in fat-only images and a subsequent 3D volumetric EAT model. Assessment of the left atrial volume index (LAVi) using a contrast-enhanced angiography*,* revealing the left atrium, its associated pulmonary veins (PV), and the left atrial appendage (LAA). Estimated volumes are indexed for body surface area. Predicted vs observed LVZ status using the combined four-variable prediction model, with two proposed risk stratification cutoffs: a 0.25 and 0.8 predicted probability is displayed

Integration of CMR imaging variables with patient characteristics in a routine clinical setting is feasible and allows to estimate individual patients’ LVZ risk (Fig. 6).

Treatment of AF patients requires a multimodal approach aiming not only at rhythm control via ablation, but at treatment of modifiable risk factors to influence the phenotype of epicardial adipose tissue, taking into account its role in inflammatory remodeling of the left atrium. Future treatment strategies might additionally adopt a specific molecular approach with a direct intervention in the inflammatory pathway and promote “browning” of the epicardial adipose tissue. Presently, it remains unclear if any intervention, including ablation techniques, may result in a reverse remodeling of left atrial cardiomyopathy and how such an assessment should be performed. One might hypothesize that interventions that influence the phenotypic shift of epicardial adipose tissue towards the anti-inflammatory phenotype, leading to a reduction of EAT volume, could to some extent reverse proarrhythmogenic changes of the left atrial myocardium. Whether this might express itself in a reduction of LVZ on the left atrial wall remains an open question. Currently, our understanding assumes that LVZ represent parts of the left atrial wall that have irreversibly changed into fibrotic tissue. This assumption might not take into account the full picture of underlying inflammatory processes influencing electrical signal generation and conduction. Recent evidence is challenging the current paradigm, showing that LVZ display dynamic changes with the possibility of regression after successful AF ablation with a decrease in AF burden [51].

This research highlights the value of including an epicardial adipose tissue assessment into prediction models. The proposed prediction model using clinical parameters (age and gender) combined with a CMR-based assessment of LAVi and LA-EATVi showed a good prediction of LVZ in the studied patient cohort. With the selection of a lower predicted probability cutoff at 0.25, the high negative predictive value is especially useful for the identification of patients with a low risk of left atrial fibrosis. This approach could be applied to identify patients assumed to be without or in an early stage of LA cardiomyopathy.

The pre-procedural identification of patients with low risk of atrial cardiomyopathy for single-shot ablation procedures like cryo-balloon or pulsed field ablation impacts current clinical routine. Patients not considered low risk by validated prediction models may need to undergo electroanatomic mapping with the possibility for an extended radiofrequency ablation approach upon identification of LVZ. The extended ablation (atrial substrate modification) beyond isolation of the pulmonary vein ostia aims to electrically isolate areas of fibrotic myocardium, which may lead to improved ablation outcome [38]. The current work suggests that inclusion of left atrial adipose tissue volume may be part of such a prediction model. Whether this proposed patient selection algorithm translates into better ablation outcomes in the long term remains to be investigated.

This work encourages further investigation of epicardial adipose tissue in connection with AF treatment. Taking into account the progress in research regarding AF pathophysiology, emphasizing the role of epicardial adipose tissue as an important contributor to inflammation-mediated LA remodeling, it becomes clear to ramp up efforts to influence this key player by intensified treatment of cardiovascular risk factors and comorbidities. The newly available drug classes of GLP-1 analogs and SGLT-2 inhibitors influence epicardial adipose tissue by directly promoting a phenotypic shift towards an anti-inflammatory state and will be of particular interest in AF treatment [24, 52–54].

## Limitations

This study is a single-center, single-arm correlative study with a relatively small patient cohort, and we do not provide data on clinical follow-up. Patients with re-do ablation procedures were excluded because these patients were likely in a more advanced stage of left atrial cardiomyopathy. Furthermore, low-voltage zone mapping in re-do cases may reflect ablation scars, confounding primary fibrosis assessment. Due to the small sample size, we were not able to demonstrate a statistically significant difference with regard to AF type and the presence of LVZ. This is in disagreement with our previous study including a large cohort [44]. The area of LVZ as a measure of LA fibrosis extent has not been routinely assessed; therefore, no classification of patients by severity of LA fibrosis was possible. Adipose tissue extent was calculated from 2D images using a summation of slices technique. To cover the entire left atrium, slice thickness and spacing were chosen at the discretion of the operator and may therefore vary between scans of the study population. The observed good absolute agreement of LA-EATV assessment between raters suggests suitable reliability for group-level analyses. The results should be interpreted with caution for individual-level decision making, as the wide confidence interval reflects substantial measurement variability. For clinical decision making, a high reliability threshold (ICC > 0.90) is recommended. Furthermore, left atrial volume estimation was performed from a non-ECG gated angiography providing a mean volume between systole and diastole. This does not represent the true maximum LA volume.

While our results support the assumption of low multicollinearity, we acknowledge that the relatively small sample size limits the precision of these estimates. It is noteworthy that while bootstrap validation confirmed the significance of LA-EATVi and gender as key predictors, next to age and LAVi, the 95% BCa confidence intervals for their coefficients included zero. This finding underscores statistical uncertainty related to our modest sample size and emphasizes the importance of external validation to confirm these results before clinical application.

## Supplementary Information

Below is the link to the electronic supplementary material.

Supplementary file1 (DOCX 433 KB)

## Data Availability

The data that support the findings of this study are not openly available due to reasons of sensitivity and are available from the corresponding author upon reasonable request.
