# Supplementary material for: Left atrial epicardial adipose tissue volume quantification by CMR aids identification of patients at low risk for left atrial cardiomyopathy
Source: Clin Res Cardiol. 2025 Sep 3;115(6):980–91. doi: 10.1007/s00392-025-02718-0 (PMC13161293; doi:10.1007/s00392-025-02718-0)
Supplement: Supplementary file 1 — Supplementary file1 (DOCX 433 KB) [file 392_2025_2718_MOESM1_ESM.docx]

**Supplement 1**

1. **Segmentation workflow for anatomical structures from CMR imaging**

The following workflow was applied to obtain the desired models of the left atrium and epicardial adipose tissue structures:

1. Import of patient DICOM files (LA-Angiography images and adipose tissue-only images from DIXON fat-water separation sequence) into the ADAS 3D software (Galgo Medical S. L, 2019)^1^:
   1. The image series were aligned using an automatic software algorithm to be able to generate models with correct reference to each other in 3D. In some cases, a manual alignment correction was necessary.
2. Creation of 3D models of anatomical structures and epicardial adipose tissue surrounding the left atrium using the segmentation tools.
   1. Left atrial model:
      1. Using the heart anatomy extraction workflow of ADAS 3D and the provided threshold method, image intensity levels were individually chosen to allow selection of contrast enhanced structures. The used MR-angiography images, obtained after application of intravenous contrast, provide a visualization of the blood perfused heart chambers and vessels. A 3D model of all contrast enhanced structures was generated through a volume rendering algorithm of the software.
      2. The region of interest was defined by extrusion of peripheral anatomical structures in the 3D model.
      3. Regional segmentation of the cardiac model was performed by automatic annotation using seeds placed on cardiac landmarks on the image slices (left ventricle, left atrium, pulmonary artery, aorta), which allows for automatic subdivision (using a selection growth algorithm) into single cardiac structures.
      4. Manual correction of the automatic structure annotation by use of paint tools consisting of a variable 3-dimensional sphere was performed if necessary.
      5. Pulmonary veins and left atrial appendage were manually annotated due to their relatively small size and complex localization.
      6. Generation of the resulting 3D left atrial model, separated from the pulmonary veins and left atrial appendage at the respective ostia.
   2. Left atrial epicardial adipose tissue model:
      1. Using the heart anatomy extraction workflow of ADAS 3D and the provided threshold method, intensity levels were selected individually to allow for selection of enhanced structures in the image. The used “Fat- Water Separation Using Graph Cut” sequence^2^ generates images, which provide a enhancement of the adipose tissue. A 3D model was generated through a volume rendering algorithm of the software.
      2. The region of interest was defined by extrusion of peripheral adipose tissue structures in the 3D model.
      3. Manual annotation of epicardial adipose tissue located in the direct vicinity of the left atrium was performed on each short axis image slice using paint tools, taking into account the pericardial border.
      4. A final 3D left atrial epicardial adipose tissue model was generated.
3. Volume quantification of generated structures:
   1. Model volumes and surface area were obtained after calculation by internal algorithms from the ADAS 3D software, which is a licensed medical product.
   2. Model data was exported and saved in the Visualization Tool Kit (VTK) format^3^, which can be used for further analysis, modification or visualization with electroanatomic mapping software during the AF ablation procedure (*Figure S1*).


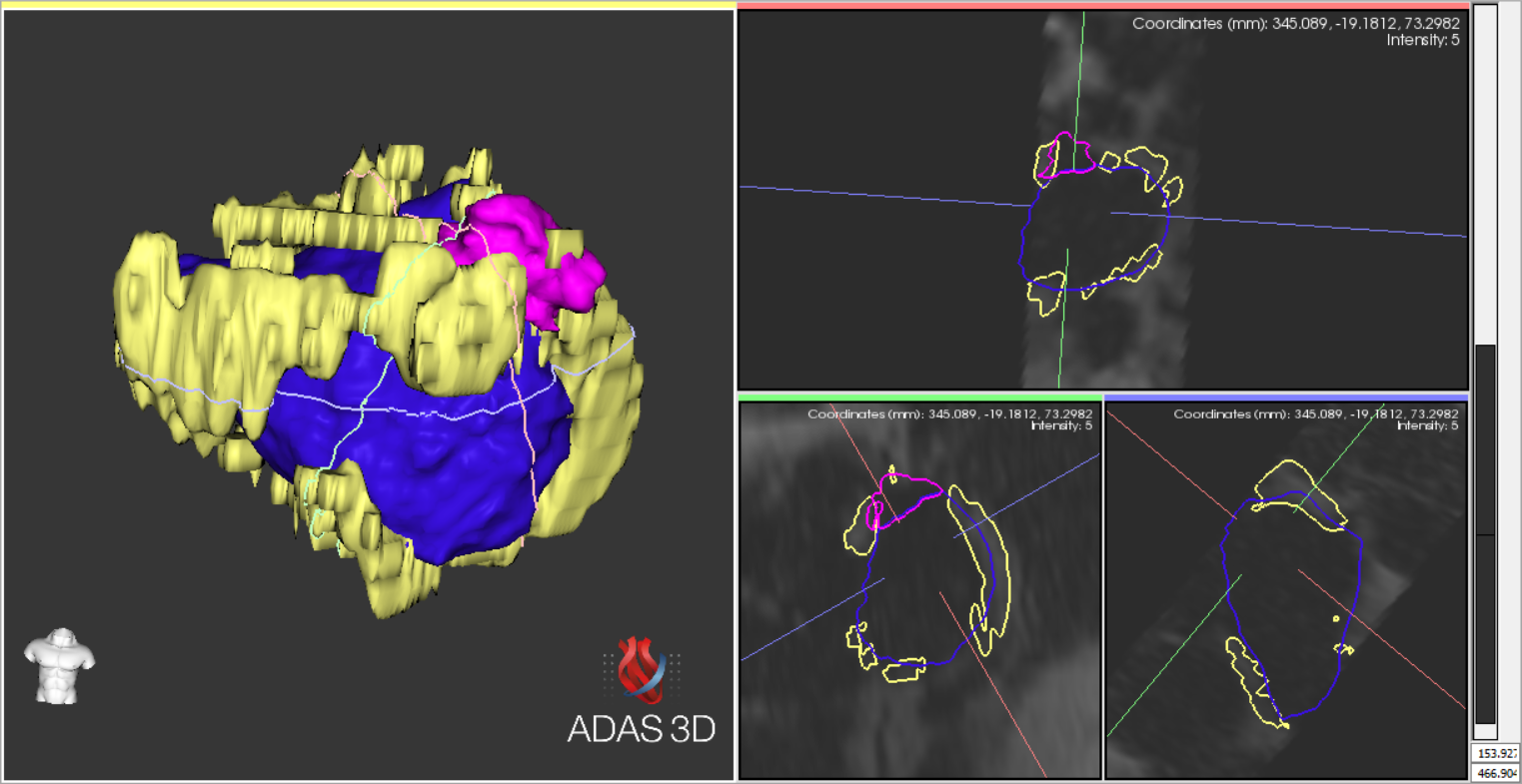


**Figure S1.** Example of a 3D model of the left atrium (LA) with epicardial adipose tissue (EAT). Segmentation of the LA 3D model was done from CMR angiography images of the LA. EAT segmentation was performed based on DIXON-based fat-water separation imaging. Left atrium (blue), left atrial appendage (pink), EAT (yellow).

1. **Results from statistical analysis**
   1. **Prediction model validation**

For the validation of the combined LVZ prediction model, bootstrap resampling was used as a method of internal validation. The aim was to assess the accuracy of the coefficients assigned to each variable in the regression equation, using a new sample of data generated from the already present data set. Results are displayed for univariate regression models (Table S1) and the multivariate regression model (Table S2).

| Variables | β coefficient | p-value in regression analysis | p-value in bootstrap validation | BCa 95%-Confidence Interval | |
| --- | --- | --- | --- | --- | --- |
| Age | 0.124 | 0.002 | 0.001 | 0.054 | 0.257 |
| Gender (female) | 1.877 | 0.002 | 0.002 | 0.645 | 3.612 |
| LAVi | 0.055 | 0.003 | 0.001 | 0.017 | 0.124 |
| LA-EATVi | 0.096 | 0.008 | 0.006 | 0.025 | 0.203 |

**Table S1.** Bootstrap validation of univariate regression models (n =2000 resamples) showing bias-corrected and accelerated (BCa) 95% confidence intervals for each predictor’s regression coefficient (β)

|  | Variables | β coefficient | p-value in regression analysis | p-value in bootstrap validation | BCa 95%-Confidence Interval | |
| --- | --- | --- | --- | --- | --- | --- |
| Combined prediction model | Age | 0.078 | 0.186 | 0.193 | -0.110 | 0.483 |
|  | Gender (female) | 2.280 | 0.014 | 0.001 | -0.121 | 7109.7 |
|  | LAVi | 0.050 | 0.061 | 0.050 | -0.025 | 8.863 |
|  | LA-EATVi | 0.120 | 0.013 | 0.001 | -0.004 | 20.06 |

**Table S2.** Bootstrap validation of the multivariate LVZ prediction model (n =2000 resamples). Bias-corrected and accelerated (BCa) 95% confidence intervals are provided for regression coefficients (β) of age, gender, left atrial volume index (LAVi), and left atrial epicardial adipose tissue volume index (LA-EATVi).

Internal validation via 2000 bootstrap resamples confirmed that each variable in the univariate models retained statistical significance, with relatively narrow bias-corrected and accelerated (BCa) confidence intervals around their regression coefficients. In the multivariate model—including age, gender, LAVi, and LA-EATVi—gender and LA-EATVi remained highly significant, while age and LAVi were significant when considering a higher boundary for statistical significance (p <0.2). However, all four coefficients exhibited wide BCa intervals that crossed zero at the lower bound, indicating imprecision in this sample. These findings suggest that the multivariate coefficient estimates should be interpreted cautiously and warrant re-estimation in a larger cohort.

- 1. **Summary table of multivariable prediction models**

| Variable combinations | β coefficient | p-value | OR | G² | R² | AICc | AUC | Sensitivity % | Specificity % |
| --- | --- | --- | --- | --- | --- | --- | --- | --- | --- |
| Age | 0.10 | 0.02 | 1.10 | 16.6 | 0.34 | 64.6 | 0.81* | 50 | 86.8 |
| Gender (female) | 1.29 | 0.05 | 3.60 |  |  |  |  |  |  |
| Age | 0.11 | 0.01 | 1.12 | 18.7 | 0.38 | 62.5 | 0.81* | 55 | 81.6 |
| LAVi | 0.05 | 0.02 | 1.05 |  |  |  |  |  |  |
| Gender (female) | 2.19 | <0.01 | 8.97 | 21.8 | 0.43 | 59.3 | 0.84* | 70 | 92.1 |
| LAVi | 0.07 | <0.01 | 1.07 |  |  |  |  |  |  |
| Age | 0.14 | <0.01 | 1.15 | 21.5 | 0.43 | 59.7 | 0.84* | 65 | 89.5 |
| LA-EATVi | 0.10 | <0.01 | 1.11 |  |  |  |  |  |  |
| Gender (female) | 2.61 | <0.01 | 13.55 | 22.9 | 0.45 | 58.3 | 0.85* | 55 | 86.8 |
| LA-EATVi | 0.14 | <0.01 | 1.15 |  |  |  |  |  |  |
| LAVi | 0.05 | <0.01 | 1.05 | 16.8 | 0.35 | 64.4 | 0.81* | 50 | 89.5 |
| LA-EATVi | 0.09 | 0.02 | 1.08 |  |  |  |  |  |  |
| Age | 0.12 | 0.01 | 1.13 | 24.8 | 0.48 | 58.7 | 0.85* | 75 | 89.5 |
| LAVi | 0.04 | 0.09 | 1.04 |  |  |  |  |  |  |
| LA-EATVi | 0.09 | 0.02 | 1.10 |  |  |  |  |  |  |
| Gender (female) | 2.76 | <0.01 | 15.87 | 30.4 | 0.56 | 53.1 | 0.89* | 70 | 89.5 |
| LAVi | 0.06 | 0.02 | 1.06 |  |  |  |  |  |  |
| LA-EATVi | 0.13 | 0.01 | 1.13 |  |  |  |  |  |  |
| Age | 0.07 | 0.14 | 1.08 | 29.8 | 0.49 | 66.5 | 0.87* | 76 | 82.9 |
| Gender (female) | 1.73 | 0.03 | 5.65 |  |  |  |  |  |  |
| LAVi | 0.06 | 0.01 | 1.06 |  |  |  |  |  |  |
| Age | 0.11 | 0.04 | 1.12 | 28.0 | 0.53 | 55.5 | 0.89* | 70 | 86.8 |
| Gender (female) | 1.99 | 0.02 | 7.30 |  |  |  |  |  |  |
| LA-EATVi | 0.13 | <0.01 | 1.14 |  |  |  |  |  |  |
| Age | 0.08 | 0.19 | 1.08 | 32.2 | 0.59 | 53.6 | 0.91* | 70 | 92.1 |
| Gender (female) | 2.28 | 0.01 | 9.78 |  |  |  |  |  |  |
| LAVi | 0.05 | 0.06 | 1.05 |  |  |  |  |  |  |
| LA-EATVi | 0.12 | 0.01 | 1.13 |  |  |  |  |  |  |

**Table S3. Multivariate LVZ prediction models:** β coefficients (p ≤ 0.20) from binomial regression, with calibration assessed by G² (log-likelihood ratio), variance explained by Nagelkerke’s R², model quality by corrected AIC (AICc), and discrimination by AUC (for all models p < 0.001). A 0.5 probability threshold was applied. Abbreviations: LAVi, left atrial volume index; LA-EATVi, left atrial epicardial adipose tissue volume index; OR, odds ratio.

1. **References**

1. Galgo Medical S. L. Instrucions for use Invesigational-ADAS_3D_v2.4. In: ; 2019.

2. Xiaoming B. Fat-Water Separation Using Graph Cut. In: SIEMENS Healthcare; 2017.

3. Schroeder W, Martin KM, Lorensen WE. *The Visualization Toolkit an Object-Oriented Approach to 3D Graphics*. Prentice-Hall, Inc.; 1998.

4. Steyerberg EW. *Clinical Prediction Models*. Springer New York; 2009. doi:10.1007/978-0-387-77244-8

5. Hosmer DW, Lemeshow S, Sturdivant RX. *Applied Logistic Regression*. 1st ed. Wiley; 2013. doi:10.1002/9781118548387
